# Incomplete Protection by Maternally Derived Antibody Against H9N2 Low-Pathogenic Avian Influenza in Commercial Broiler Chickens

**DOI:** 10.3390/ani16213505

**Published:** 2026-11-06

**Authors:** Jong-Yeol Park, In-Kyu Lee, Ji-Eun Son, Ki-Woong Kim, Kai-Qiong Zheng, Cheng-Dong Yu, Won-Bin Jeon, Hyung-Kwan Jang, Bai Wei, Min Kang

**Affiliations:** 1 Department of Avian Diseases, College of Veterinary Medicine and Center for Avian Disease, Jeonbuk National University, Iksan 54596, Republic of Korea; 2 Bio Disease Control (BIOD) Co., Ltd., Iksan 54596, Republic of Korea; inkyu.biod@gmail.com

**Keywords:** low pathogenic avian influenza, H9N2, maternally derived antibody, transfer efficiency, antibody decay, broiler chicken, Y280 lineage

## Abstract

H9N2 low-pathogenic avian influenza remains endemic in Korean poultry production, where commercial broilers rely largely on maternally derived antibodies (MDA) transferred from vaccinated breeder hens. This study quantified MDA transfer and decay and evaluated protection against homologous Y280-lineage H9N2 challenge. Paired sera were collected from breeder hens and day-old progeny on six commercial farms. MDA decay was monitored in 90 broilers from 1 to 22 days of age, and 32 birds with individual HI titers of 0, 2, 4, or 6 log_2_ were challenged intranasally at 7 days of age. Transfer efficiency averaged 18.2 ± 11.5%, and MDA had an estimated half-life of 3.2 days. Clinical signs occurred in 8/8, 8/8, 7/8, and 0/8 birds in the MDA 0, 2, 4, and 6 groups, respectively. Cecal tonsil virus detection decreased from 87.5% in the MDA 0 group to 37.5% in the MDA 6 group, although the pairwise difference was not statistically significant (*p* = 0.119). Oropharyngeal shedding occurred in all challenged birds at comparable titers. These findings suggest that higher MDA levels are associated with reduced clinical disease and lower cecal tonsil virus detection but do not prevent oropharyngeal shedding under the conditions tested.

## Simple Summary

H9N2 low-pathogenic avian influenza (H9N2 LPAIV) is endemic in Korean poultry production. Because commercial broilers have a short production cycle, they are generally not vaccinated against H9N2 and instead rely on maternally derived antibodies (MDA) transferred from vaccinated breeder hens. However, the amount of antibody transferred, its persistence, and its ability to protect chicks against infection have not been evaluated together in a single commercial system. We examined MDA transfer in six commercial broiler-breeder farms, monitored antibody decay in broilers, and evaluated protection after experimental challenge with a Y280-lineage H9N2 virus. Chicks received an average of 18.2% of the antibody level measured in their breeder hens. MDA declined rapidly, with a half-life of 3.2 days, and the population mean fell below the 6 log_2_ HI benchmark at approximately 3.2 days of age. Following challenge, higher MDA levels were associated with fewer clinical signs and reduced cecal tonsil infection. However, all challenged birds shed the virus from the oropharynx, regardless of MDA levels. These findings indicate that maternal antibodies provide short-lived and incomplete protection against H9N2 in commercial broilers and do not prevent respiratory virus shedding. Therefore, evaluation of H9N2 control strategies should consider virological endpoints in addition to clinical protection.

## 1. Introduction

Avian influenza viruses (AIV) are classified under the genus Alphainfluenzavirus of the Orthomyxoviridae family. These viruses have eight segments of negative-sense single-stranded RNA that encode at least ten proteins. Subtypes are classified based on the antigenic characteristics of two surface glycoproteins: hemagglutinin (HA) and neuraminidase (NA) [1,2]. In poultry, the pathotype is influenced by the HA cleavage sites. Highly pathogenic avian influenza viruses (HPAIV), predominantly of the H5 and H7 subtypes, feature a polybasic cleavage motif that is processed by furin-like proteases found throughout the body, allowing systemic replication of the virus. In contrast, low-pathogenic avian influenza viruses (LPAIV) mainly replicate in the respiratory and digestive tracts [3].

Although H9N2 is classified as an LPAIV, the economic losses associated with it are considerably greater than this classification would suggest because the virus is endemic in many regions and is frequently involved in mixed infections. In Korea, H9N2 was first detected in 1996 and represented by the isolate A/chicken/Korea/96006/96, which was genetically related to the waterfowl-origin virus A/duck/Hong Kong/Y439/97 [4,5]. Three Eurasian lineages are currently recognized: G1, Y280, and Korean/Y439 [4], and viruses of the Y280 lineage have predominated in Korean poultry in recent years [6,7]. Under field conditions, natural co-infection of commercial broilers with infectious bronchitis virus (IBV) and Mycoplasma gallisepticum has been associated with high mortality [8]. Experimental co-infection with QX genotype IBV increased mortality and suppressed growth [9]. Concurrent H9N2 infection reduced the minimum infectious dose of velogenic Newcastle disease virus [10]. Co-infection with Ornithobacterium rhinotracheale aggravated respiratory disease [11]. This lineage is also of public health relevance, as Y280/G9 viruses have repeatedly donated internal gene segments to human-pathogenic reassortants, such as H7N9, H5N6, H10N8, and H5N1 [4,12], and account for the majority of documented human H9N2 infections [13,14].

In Korea, vaccination against H9N2 is administered to breeder and layer flocks but not to commercial broilers, in which the production cycle of 28–35 days renders the individual administration of inactivated vaccines impractical for both operational and economic reasons. Therefore, the protection of broilers is assumed to be conferred by maternally derived antibody (MDA) transferred from vaccinated breeder herds. This assumption rests on three requirements: that a substantial proportion of the antibody present in the breeder hen is transferred to the chick, that the transferred antibody persists over a meaningful part of the production cycle, and that the titers attained are protective against the circulating viral lineage.

Each of these requirements has been examined individually but not within a single production system. The transfer efficiency for AIV has been reported as 19.5% in a commercial broiler-breeder flock in which antibodies were measured directly in the hens and their day-old progeny [15], and as 60.35% in an Iranian cohort in which the progeny were sampled at 3 days of age and the breeder titers were derived from farm records [16]; this threefold discrepancy remains unresolved. The decay of maternal antibodies to AIV has been estimated to have a half-life of 4.2 days in a study in which ten poultry pathogens were monitored within the same cohort [17]. However, it has not been specifically determined for H9N2 by hemagglutination inhibition (HI) in a Y280-endemic system. The relationship between MDA titer and protection is the least well-characterized of the three. It has been established that MDA suppresses the humoral response to inactivated H9N2 vaccine under field conditions [18], that commercial inactivated vaccines fail to prevent infection and shedding in the presence of MDA [19], and that vaccination pressure accelerates the antigenic drift of circulating H9N2 [20]. However, protection has not been graded across MDA strata within a single challenge experiment, with outcomes reported separately for each anatomical site.

This last consideration merits particular attention. Maternal immunity in chicks differs in composition from actively acquired immunity, since chicks receive approximately 30% of the immunoglobulin Y (IgY) present in the dam, but less than 1% of her IgA and IgM [21]. Because IgA rather than IgY is the isotype that limits influenza virus replication at the upper respiratory mucosa [22,23], maternal antibodies may be expected to afford uneven protection at different anatomical sites. This distinction is of practical importance, as transmission is determined by respiratory shedding rather than clinical disease.

Therefore, the present study aimed not only to estimate the transfer efficiency and decay of MDA in commercial broilers under Korean field conditions, but also to determine the MDA titer required for protection against a homologous Y280-lineage challenge and quantify the effect of MDA on clinical disease, respiratory shedding, and enteric infection separately.

## 2. Materials and Methods

### 2.1. Ethical Statement and Housing

All animal procedures were reviewed and approved by the Institutional Animal Care and Use Committee (IACUC) of Jeonbuk National University (approval number: NON2024-198). The animal facility of Jeonbuk National University was fully accredited by the National Association of Laboratory Animal Care (NALAC). To minimize potential confounding attributable to environmental and housing conditions, commercial Arbor Acres broilers were housed in Biosafety Level 2 (BSL-2) isolators under controlled conditions of temperature, humidity, and lighting, and feed and water were provided ad libitum. The reporting complies with the ARRIVE 2.0 guidelines [24].

### 2.2. Field Serological Survey

Paired serum samples were collected from breeder hens and their day-old progeny at six commercial broiler-breeder farms in Jeonbuk Province, Republic of Korea. All six farms operated naturally ventilated, open-sided houses under the management standards of a single supplying integrator. Between 9 and 15 hens were bled per farm, and the flock age at the time of sampling ranged from 30 to 43 weeks. The progeny were bled at hatch and originated from eggs of the same flocks. The H9N2 vaccination history of the breeder flocks was not available to the investigators and was therefore treated as an unmeasured source of between-farm variation.

### 2.3. Challenge Virus

The Y280-lineage H9N2 LPAIV isolate A/chicken/Korea/A21-MRA-003/2021 was used for the challenge experiment [25]. The virus was propagated in 10-day-old specific pathogen-free (SPF) embryonated chicken eggs according to standard procedures and titrated using the Reed–Muench method.

### 2.4. Serology

Blood was centrifuged at 15,000× *g* for 3 min, and the serum was stored at −20 °C until testing. Antibodies to H9N2 were quantified using the HI test performed according to a standard procedure [26], using two-fold serial dilutions of serum and four hemagglutinating units of homologous antigen. Titers were expressed as log_2_ values of the reciprocal of the highest dilution producing complete inhibition. Positive, negative, antigen, and erythrocyte controls were included on every plate.

### 2.5. Estimation of Maternal Antibody Transfer Efficiency

Transfer efficiency was calculated for each farm on a linear scale rather than the log_2_ antibody scale, as the ratio of the geometric mean titer (GMT) of the day-old progeny to that of the corresponding breeder flock:

(1) Transfer efficiency (%) = 100 × 2^(progeny log_2_ GMT − breeder log_2_ GMT)^

This convention is used in comparable literature [15,16] and was retained in this study to permit direct comparison. Farm-level values were summarized as the arithmetic mean ± standard deviation across the six farms, and a pooled estimate derived from the mean log_2_ decrement was reported.

### 2.6. Maternal Antibody Decay

HI titers were measured serially in 90 commercial broilers from 1 day of age at 7-day intervals until 22 days of age. The titer was modelled as a log-linear function of age, and the slope was converted to a half-life as t½ = −1/slope on the log_2_ scale. The ages at which the fitted population mean crossed 6, 4, and 2 log_2_ were read from the fitted line. An HI titer of 6 log_2_ was used as the principal reference threshold, as this titer represents the efficacy criterion applied to H9N2 LPAI vaccines in the Republic of Korea [26].

### 2.7. Challenge Experiment

Following arrival at the animal facility, the birds were acclimatized for 6 days before the challenge experiment. Thirty-two commercial Arbor Acres broilers were screened for H9-specific antibodies using the HI test at 7 days of age. Based on their individual pre-challenge HI titers, birds with titers of exactly 0, 2, 4, or 6 log_2_ were as-signed to the corresponding MDA 0, MDA 2, MDA 4, or MDA 6 group, respectively, with eight birds in each group. Group allocation was based on individual HI titers rather than randomized allocation. The MDA 0 group served as the antibody-negative challenge control. Clinical signs were assessed without blinding to group allocation. Because allocation was based on the measured titer and not performed randomly, the design constituted a stratified comparison of naturally acquired antibody levels rather than a randomized experiment.

On the same day, all 32 birds were intranasally challenged with 10^7.0^ EID_50_ of A21-MRA-003 in 0.2 mL. No mock-challenged group receiving phosphate-buffered saline (PBS) was included. Accordingly, the MDA 0 group represented a challenged group with no detectable HI antibody rather than an uninfected negative control. The birds were observed daily for 5 days post-challenge (DPC) for mortality and clinical signs, which comprised depression, ruffled feathers, respiratory signs, and reduced feed intake. At 5 DPC, oropharyngeal (OP) swabs were collected from all birds, which were then euthanized, and the cecal tonsil (CT) was collected.

### 2.8. Virus Isolation and Titration

No predefined inclusion or exclusion criteria were applied. All virus titer data obtained from CT, and OP swab samples were included in the analyses, with no animals or data points excluded. OP swabs and CT samples were homogenized in phosphate-buffered saline supplemented with antibiotics, clarified by centrifugation, and inoculated into 10-day-old specific pathogen-free (SPF) embryonated chicken eggs. Allantoic fluid was harvested after incubation and examined for hemagglutination activity. Titers were calculated using the Reed–Muench method and expressed as log_10_ EID_50_/mL values. Samples in which no hemagglutinating activity was detected after passage were considered negative.

### 2.9. Protection Index and Absolute Risk Difference

The protection index (PI) was calculated as the relative reduction in the proportion of CT virus-positive birds in each MDA group compared with the MDA 0 group. The absolute risk difference (ARD) was calculated as the difference in CT virus positivity between each MDA group and the MDA 0 group, expressed in percentage points.

The PI and ARD were calculated using the following equations:

PI (%) = 100 × [(positive rate in the MDA 0 group − positive rate in the test group)/positive rate in the MDA 0 group]

ARD (percentage points) = 100 × [positive rate in the test group − positive rate in the MDA 0 group]

The confidence intervals for the PI were derived from the corresponding risk ratios.

### 2.10. Statistical Analysis

Continuous variables, namely HI titers and viral titers, were compared using one-way analysis of variance (ANOVA) with Tukey’s multiple comparison test after normality and homogeneity of variance were confirmed. Binary variables, namely the presence of clinical signs and virus isolation, were compared using Fisher’s exact test, and the ordered relationship between the MDA stratum and each binary outcome was examined using the Cochran–Armitage test for trend, using the log_2_ titer as the score. Exact (Clopper–Pearson) 95% confidence intervals are reported for all proportions. The relationship between the breeder titer and transfer efficiency was assessed using Pearson’s correlation. Statistical computations were performed using SPSS version 19.0 (IBM Corp., Armonk, NY, USA) and Python 3 with SciPy 1.17. Differences were considered significant at *p* < 0.05, two-sided. With eight birds per group, the study had approximately 40% statistical power to detect the observed difference in CT positivity between the extreme strata; the trend test across the ordered strata was therefore treated as the primary inferential procedure.

### 2.11. Data Visualization and Graphical Editing

Data were analyzed and plotted using GraphPad Prism 5 (GraphPad Software, San Diego, CA, USA). Claude (Anthropic, San Francisco, CA, USA) and Scispace (PubGenius Inc, Milpitas CA, USA) were used to assist with the graphical editing and refinement of figures. All generative artificial intelligence-assisted graphical content was reviewed and verified by the authors, who take full responsibility for the accuracy and integrity of the final figures.

## 3. Results

### 3.1. Maternal Antibody Titers in Breeder Flocks and Their Day-Old Progeny

**Figure 1:**
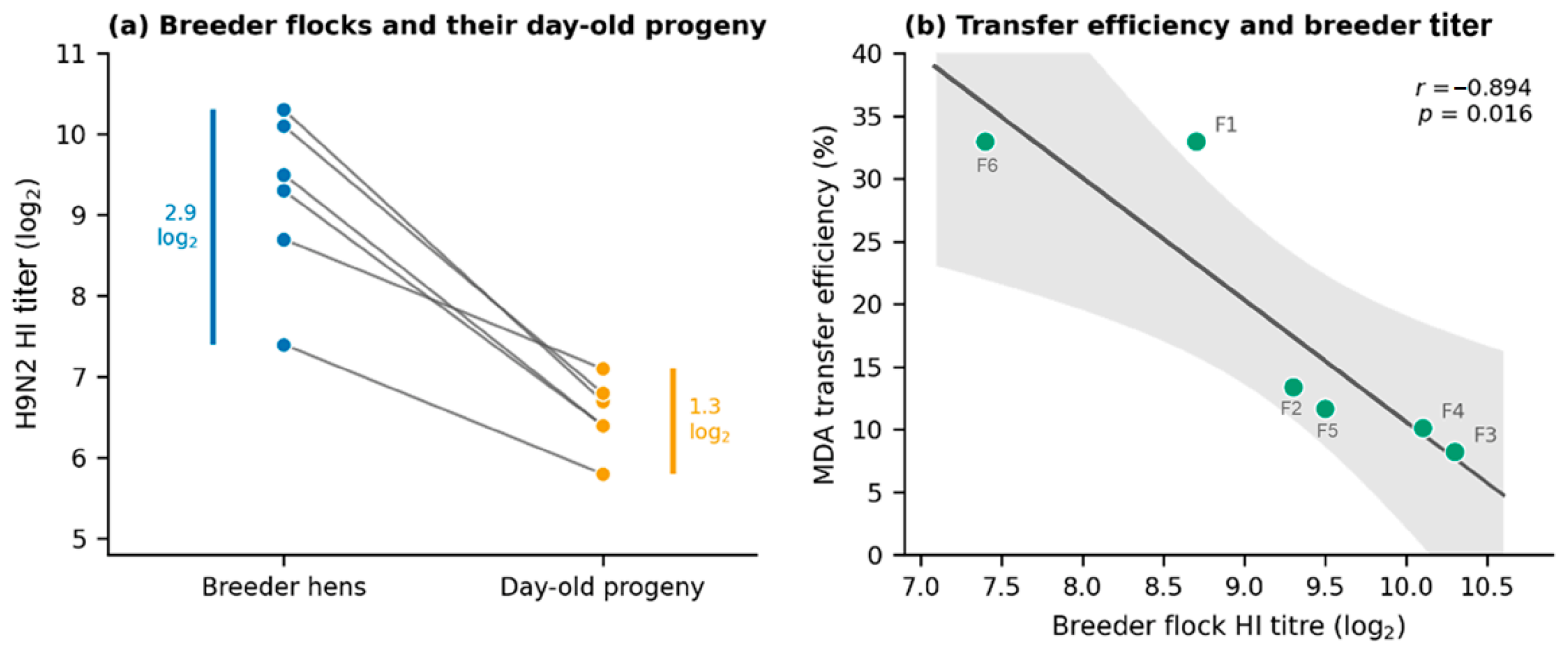
Hemagglutination inhibition (HI) antibody titers to H9N2 in six commercial broiler-breeder flocks and their day-old progeny. (**a**) Paired titers of each breeder flock and its progeny. The vertical bars denote the between-farm range, which was 2.9 log_2_ in the hens and 1.3 log_2_ in the progeny. (**b**) Transfer efficiency plotted against breeder flock titer, together with the fitted regression line and its 95% confidence band (r = −0.894, *p* = 0.016). Because transfer efficiency is calculated as a function of breeder titer, part of this relationship is structural; the compression of the progeny range shown in panel (**a**) is independent of the ratio.

The HI titers in the six breeder flocks ranged from 7.4 ± 0.8 to 10.3 ± 0.8 log_2_, with a mean of 9.2 ± 1.1 log_2_, corresponding to a nearly eightfold difference on the linear scale between the lowest and highest flock. Titers in the day-old progeny covered a considerably narrower range, from 5.8 ± 0.7 to 7.1 ± 1.7 log_2_, with a mean value of 6.7 ± 0.4 log_2_. The between-farm range was 2.9 log_2_ in hens and 1.3 log_2_ in their progeny (Table 1, Figure 1a).

The transfer efficiency ranged from 8.2% to 33.0% between farms, with an arithmetic mean of 18.2 ± 11.5% (95% confidence interval (CI) 6.1–30.4%). The pooled estimate derived from the mean log_2_ decrement of 2.68 log_2_ was 15.6%. When the two farms with the highest values were excluded (farms 1 and 6, both 33.0%), the remaining four farms fell within a narrow range of 10.9 ± 2.2%.

### 3.2. Relationship Between Breeder Titer and Transfer Efficiency

A negative correlation was observed between breeder HI titer and calculated transfer efficiency (r = −0.894, *p* = 0.016). However, because breeder titer is incorporated into the transfer-efficiency calculation, this association should be interpreted cautiously and does not independently establish a biological mechanism. Breeder titer was not a significant predictor of progeny titer (r = 0.596, *p* = 0.212). The two farms with the highest transfer efficiency of 33.0% had the lowest breeder titers, Farm 6 at 7.4 log_2_ and Farm 1 at 8.7 log_2_, whereas the farm with the highest breeder titer, Farm 3 at 10.3 log_2_, exhibited the lowest transfer efficiency of 8.2%.

Because transfer efficiency is calculated as a function of the breeder titer, part of this correlation is structural, and its magnitude should not be over-interpreted. The observation that is independent of the ratio is the compression of the progeny range relative to that of the hens described in Section 3.1, which indicates that the additional antibody present in the more strongly immunized hens was not transferred to their progeny in a proportional manner.

### 3.3. Decay of Maternal Antibody

**Figure 2:**
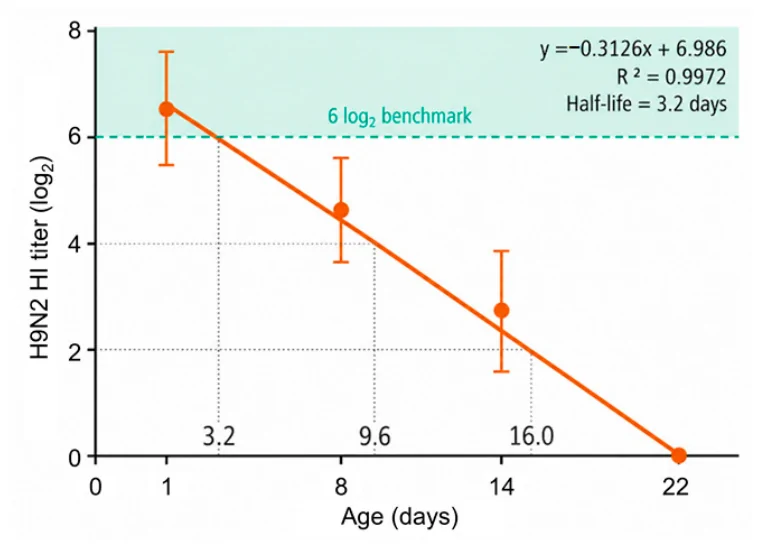
Decay of maternally derived antibodies to H9N2 in commercial broilers. HI titers were measured serially in 90 broilers between 1 and 22 days of age and are presented as mean ± standard deviation (SD). The fitted log-linear regression, with a slope of −0.30 log_2_ per day (R^2^ = 0.9972), corresponded to a half-life of 3.2 days. The shaded band denotes the 6 log_2_ HI benchmark applied in the evaluation of H9N2 LPAIV vaccines [26]; the population mean fell below this benchmark at approximately 3.2 days of age.

**Figure 3:**
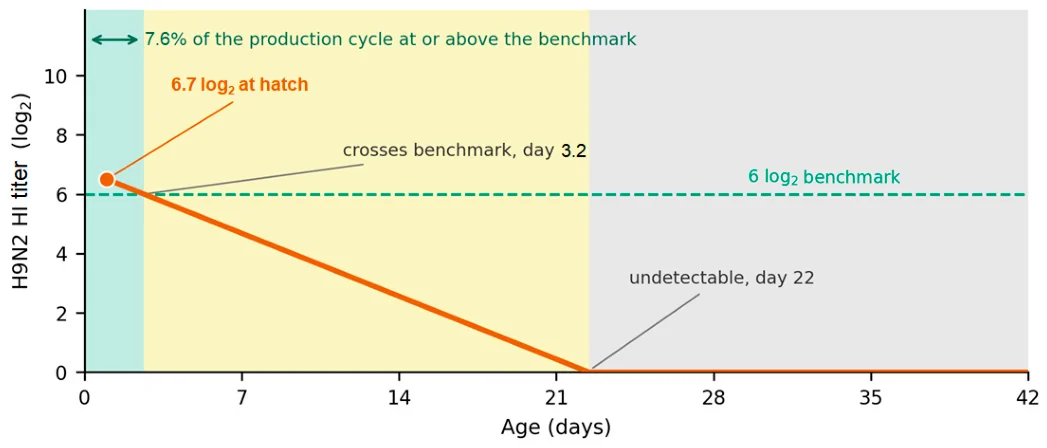
Maternally derived antibody titer in relation to the broiler production cycle. The fitted decay, with a half-life of 3.2 days, was plotted against the 6 log_2_ benchmark [27]. Decay from the mean titer of 6.7 log_2_ observed at hatch in the present study: antibody at or above the benchmark was present for approximately 3.2 days, corresponding to 7.6% of a 42-day cycle; detectable but sub-benchmark antibody was present until approximately day 22; and no maternal antibody was detectable during the remainder of the cycle.

Serial measurements in 90 broilers between 1 and 22 days of age demonstrated a log-linear decline from a mean of 6.7 ± 0.4 log_2_ at hatching (Figure 2). The fitted slope of −0.30 log_2_ per day corresponded to a half-life of 3.2 days (R^2^ = 0.9972). Relative to the 6 log_2_ HI benchmark applied in the evaluation of H9N2 LPAIV vaccines [26], the population mean fell below the benchmark at approximately 3.2 days of age, below 4 log_2_ by day 9.6, and below 2 log_2_ by day 16, and reached the limit of detection at approximately 22 days. Within a production cycle of 35–42 days, antibodies at or above the benchmark were present for less than 8% of the life of the bird, and detectable but sub-benchmark antibodies were present for approximately half of the cycle (Figure 3).

The fitted model was internally consistent with the birds used in the challenge experiments. When extrapolated to 7 days of age, the regression predicted a population mean of 4.7 log_2_, and the four titers identified by screening at that age, namely 0, 2, 4, and 6 log_2_, were distributed around that value as expected in a population exhibiting individual variation around a declining mean (Figure 3).

### 3.4. Clinical Protection Following Challenge

**Figure 4:**
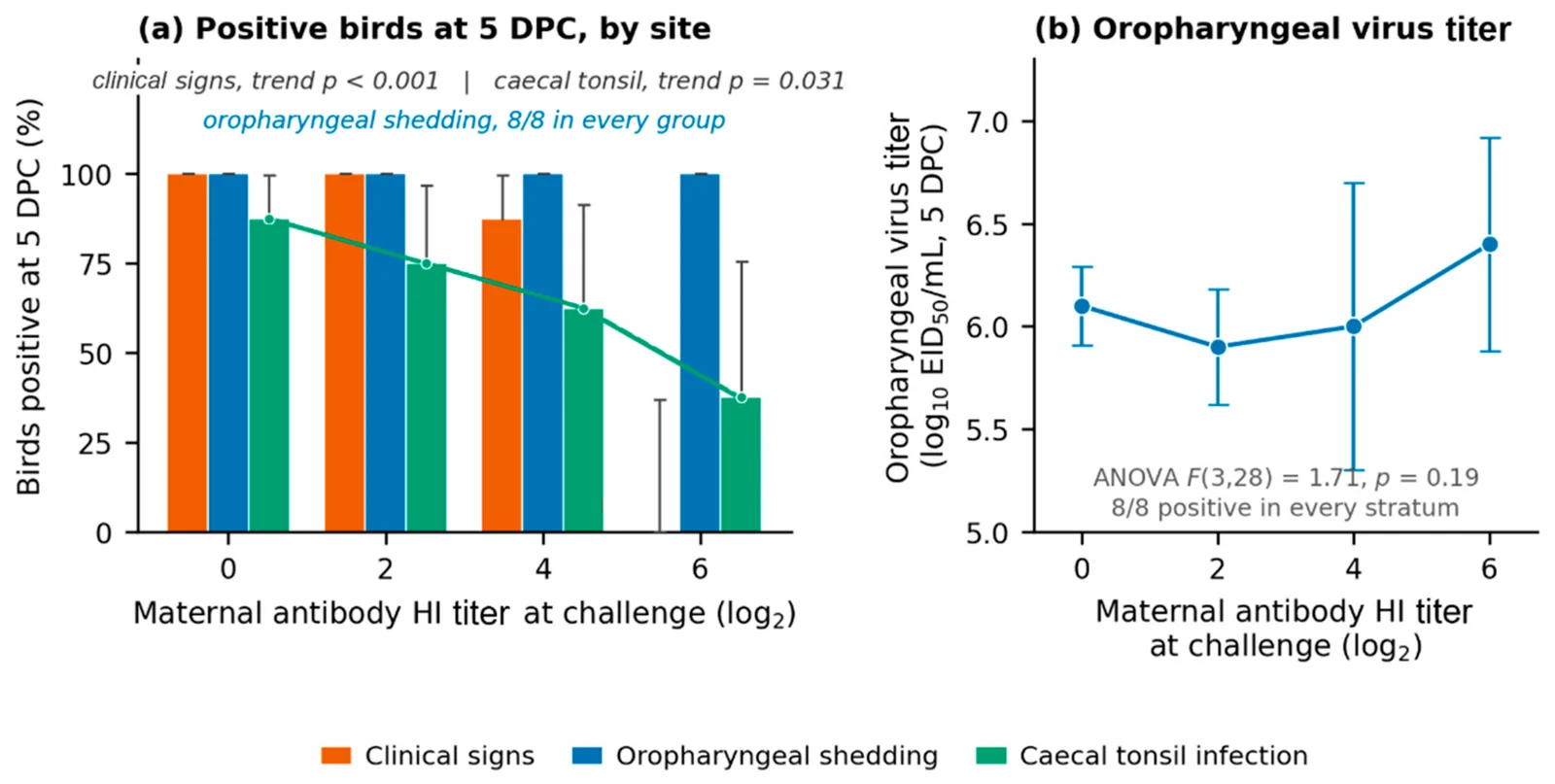
Outcome of challenge with A21-MRA-003 at 7 days of age in commercial broilers, stratified by MDA titer. (**a**) Proportion of birds positive for clinical signs, OP shedding, and CT infection 5 days post-challenge. Exact 95% confidence intervals are shown for CT infections. Clinical signs and CT infection decreased as the antibody titer increased (Cochran–Armitage test for trend, *p* < 0.001 and *p* = 0.031, respectively), whereas oropharyngeal shedding was detected in all eight birds in each group. (**b**) OP virus titer according to group, which did not differ significantly between groups (one-way ANOVA, *p* = 0.19).

Clinical signs recorded after challenge were closely related to the MDA titer present at the time of challenge (Table 2, Figure 4a). All eight birds in the MDA 0 group and all eight birds in the MDA 2 group developed clinical signs; seven of the eight birds in the MDA 4 group were affected, and no bird in the MDA 6 group exhibited any clinical sign. The trend across the ordered strata was significant (Cochran–Armitage Z = −4.40, *p* < 0.001), as was the difference between the two extreme groups (Fisher’s exact test, *p* = 0.0002). No mortality occurred in any group during the observation period.

### 3.5. Virus Shedding and Tissue Infection

Viruses were isolated from the OP swabs of all eight birds in each of the four groups at 5 DPC, including those of MDA 6 group, in which no clinical signs were observed. The mean shedding titers were 6.1 ± 0.19, 5.9 ± 0.28, 6.0 ± 0.70, and 6.4 ± 0.52 log_10_ EID_50_/mL in the MDA 0, MDA 2, MDA 4, and MDA 6 groups, respectively, and did not differ significantly between groups (F(3,28) = 1.71, *p* = 0.19); the numerically highest titer was recorded in the group with the highest MDA titer (Table 2, Figure 4b).

In contrast, the isolation of virus from CT decreased as MDA titer increased. Viruses were recovered from 7/8 (87.5%; 95% CI, 47.3–99.7%) birds in the MDA 0 group, 6/8 (75.0%) in the MDA 2 group, 5/8 (62.5%) in the MDA 4 group, and 3/8 (37.5%; 95% CI, 8.5–75.5%) in the MDA 6 group, corresponding to PI values of 14.3%, 28.6%, and 57.1%, respectively. Compared with the MDA 0 group, ARDs were −12.5, −25.0, and −50.0 percentage points in the MDA 2, MDA 4, and MDA 6 groups, respectively. The trend across the ordered strata was significant (Cochran–Armitage Z = −2.16, *p* = 0.031), but no pairwise comparison attained significance (extreme strata, Fisher’s exact test *p* = 0.119; overall χ^2^ *p* = 0.183), and the CI of each PI value included zero, that of the MDA 6 group being −8.9–83.1%. Among the birds from which the virus was recovered, the CT titer did not differ between groups (3.0–3.3 log_10_ EID_50_/mL; F(3,17) = 0.13, *p* = 0.94), suggesting that higher MDA levels were associated with a lower probability of CT virus detection rather than with reduced viral titers among CT-positive birds.

## 4. Discussion

In countries where H9N2 LPAIV is endemic, control in commercial broilers is complicated by the short production cycle, which renders the individual administration of inactivated vaccines impractical; protection is therefore commonly assumed to be conferred by antibodies transferred from vaccinated breeder flocks. However, the persistence and protective capacity have been investigated in different systems using different assays and against different virus lineages, with the consequence that the amount of protection actually available to a commercial broiler at a given age has remained uncertain.

In the present study, a field serological survey, serial monitoring of antibody decay, and an MDA-stratified challenge experiment were combined to examine the three requirements within a single commercial system exposed to the Y280 lineage. The transferred antibody decayed with a half-life of 3.2 days and fell below the 6 log_2_ benchmark within approximately 3.2 days after hatching. The protection observed after the challenge was pronounced against clinical disease, weak against enteric infection, and absent against respiratory shedding. Taken together, these findings indicate that the protection available to commercial broilers from maternal antibodies alone is limited in amount, brief in duration, and incomplete in the dimension that determines transmission.

Our estimate of transfer efficiency is in agreement with the findings reported previously by Gharaibeh et al. [15], who measured antibody directly in breeder hens and in their day-old progeny within a commercial broiler-breeder flock and obtained a value of 19.5% for AIV. It is also compatible with the report of Hamal et al. [21], in which dam-to-chick transfer of total IgY in meat-line breeders was approximately 30% when measured by enzyme-linked immunosorbent assay (ELISA), given that antigen-specific HI measures functional rather than total immunoglobulin and that their chicks were sampled at 3 days of age. The considerably higher value of 60.35% reported by Mirzaie et al. [16] (Table 3), which has frequently been adopted as the reference figure for H9N2, was obtained under two methodologically distinct conditions: the progeny were sampled at 3 days of age rather than at hatch, and, more importantly, the breeder titers were not measured contemporaneously but were reconstructed from questionnaires completed by farmers, veterinarians, and farm managers. A denominator obtained in this manner will not necessarily correspond to the antibody concentration circulating in the hens that laid the sampled eggs. Therefore, it appears that those studies in which paired samples were measured directly converge upon a value of approximately 18–30%, and that the higher figure should be applied with caution when the amount of maternal protection available to commercial broilers is estimated.

To the best of our knowledge, progeny HI titers varied over a narrower range (5.8–7.1 log_2_; span, 1.3 log_2_ units) than breeder HI titers (7.4–10.3 log_2_). This compressed range indicates that differences in breeder titers were not reflected proportionally in the observed progeny titers in this cohort. However, because breeder titer is incorporated into the transfer-efficiency calculation, the observed negative correlation should be interpreted cautiously and does not independently establish a biological mechanism limiting antibody transfer. A saturating mechanism constitutes the most parsimonious explanation, since the deposition of IgY in the yolk is receptor-mediated and finite, and the amount of antibody available to the chick is therefore limited by yolk capacity and yolk sac absorption rather than by the concentration circulating in the hen. This interpretation is consistent with the observation of Hamal et al. [21] that dam plasma IgY was a direct indicator of transfer but only within a bounded percentage and with the finding that transfer percentages for ten pathogens measured in the same birds varied by nearly twentyfold [15], a degree of variation that cannot be attributed to maternal antibody concentration alone. As noted in Section 3.2, part of the correlation reported here is arithmetically induced, and the argument therefore rests upon the compression of the progeny range rather than the correlation coefficient itself. The practical consequence is that the intensification of breeder vaccination is subject to strongly diminishing returns as a means of protecting broilers, and this consequence is reinforced by the decay data: a chick hatching at the observed mean of 6.7 log_2_ falls below the benchmark within approximately three days.

The half-life of 3.2 days obtained here lies at the lower end of the published range. Gharaibeh and Mahmoud [17] estimated the half-life of maternal antibody in commercial broiler chicks for ten pathogens within a single cohort and under a single protocol, obtaining 7.0 days for chicken anemia virus, 6.3 days for Newcastle disease virus, 5.3 days for avian encephalomyelitis virus, 5.1 days for infectious bursal disease virus, 4.9 days for Mycoplasma gallisepticum, 4.7 days for reovirus, 4.2 days for AIV, 4.1 days for Mycoplasma synoviae, 3.9 days for IBV, and 3.8 days for infectious laryngotracheitis virus (Table 4). The difference between 4.2 and 3.2 days is modest, and the two studies employed different assays, ELISA in one study and HI in the present one; therefore, the values are not strictly interchangeable; however, the ranking is informative. Maternal antibody to AIV is among the shortest-lived of those routinely monitored in broilers, and AIV is the only one of these agents for which the protection of Korean broilers depends on maternal antibody without any subsequent active vaccination. For infectious bursal disease and Newcastle disease, the rapid decay of maternal antibody is regarded as a problem of vaccination timing; for H9N2 in broilers, the same rapid decay constitutes a problem of protection with nothing to follow it.

Values from [17] were obtained by ELISA, with the exception of Newcastle disease virus, for which HI was used; the value of the present study was obtained using HI. The assays are not strictly interchangeable, and the comparison is presented to establish the ranking and approximate magnitude.

Our decay estimate is consistent with published field serology. In unvaccinated broiler flocks in Libya, seropositivity to H9N2 was 0% at 10 days of age, 22% at 24 days, and 72% at 38 days, a pattern that the authors attributed to interference by maternal antibody during the early period and to active field infection thereafter [28]. Seroconversion commencing between the second and third weeks of age is precisely what would be predicted from the decay curve reported here, in which antibody became undetectable at approximately day 22. Two independent approaches, namely serial measurement in the present study and the timing of field seroconversion in that study, therefore indicate the same period of susceptibility.

The most notable finding of the challenge experiment was that protection differed according to the anatomical site examined. Clinical signs were abolished at 6 log_2_, CT infection was approximately halved, and OP shedding was unaffected, being detected in all birds of all groups at comparable titers. These findings suggest that higher MDA levels may be associated with reduced clinical disease and a lower frequency of CT virus detection under the conditions tested, but they did not prevent OP shedding. The proportion of CT-positive birds declined from 87.5% in the MDA 0 group to 37.5% in the MDA 6 group. This 50-percentage-points may be biologically relevant, but the pairwise comparison was not statistically significant (*p* = 0.119), and the confidence interval for the protection index included zero. The significant trend across ordered MDA strata suggests a possible relationship between MDA level and CT positivity; however, the limited sample size precludes a firm conclusion regarding the magnitude of enteric protection. These findings should be considered exploratory and require confirmation in a larger study. One possible explanation for this difference is that maternally transferred antibodies may provide more effective systemic than mucosal protection. Chicks receive substantially more maternal IgY than IgA or IgM, resulting in the transfer of systemic humoral immunity with comparatively limited transfer of antibodies associated with mucosal protection [21]. At respiratory surfaces, secretory IgA is considered an important component of local immunity, while antibodies originating in serum may reach respiratory and ocular surfaces through transudation and glandular secretions [22]. Evidence from other respiratory infection models also indicates that systemic antibodies may reduce disease severity without necessarily preventing infection at the upper respiratory mucosa [23], and respiratory secretory IgA levels have been associated with viral shedding [29]. However, because immunoglobulin isotypes and mucosal antibody levels were not measured in the present study, the contribution of these mechanisms to the observed pattern remains speculative. Further studies measuring systemic and mucosal antibody responses alongside virus replication and shedding are needed to determine whether differences in antibody distribution or isotype contribute to the incomplete protection observed here.

To our knowledge, few studies have assessed clinical outcomes, CT virus detection, and OP shedding across graded MDA titer groups within a single H9N2 challenge experiment. The present findings highlight the importance of evaluating multiple outcomes when assessing MDA-associated protection. In particular, the absence of clinical signs should not be interpreted as evidence that infection or virus shedding has been prevented. Nevertheless, because all birds were challenged and no contact-transmission experiment was conducted, the present study cannot establish whether MDA levels influence bird-to-bird transmission under commercial production conditions.

This interpretation is consistent with several observations reported previously. Commercial inactivated vaccines, which elicit systemic IgY without a substantial mucosal IgA response, reduce disease but fail to prevent infection and shedding in broilers with MDA [19]. MDA suppresses the antibody response to inactivated vaccination under field conditions [18], and vectored vaccines are not automatically exempt from interference, as maternal antibodies have been shown to inhibit Newcastle disease virus-vectored avian influenza vaccine [30]. This interpretation has direct epidemiological implications for public health. Birds in the MDA 6 group were clinically normal but shed virus at 6.4 log_10_ EID_50_/mL, which was the highest titer recorded in the experiment. Therefore, maternal antibodies at this level did not reduce the infectious output of the birds but removed the clinical signs that would have prompted investigation, with the consequence that a flock evaluated on the basis of clinical signs or serology alone would be classified as protected while continuing to shed virus despite the absence of clinical signs. Because transmission was not directly evaluated, the present study cannot determine whether this shedding resulted in sustained bird-to-bird transmission.

Further consideration is given to the benchmark employed for the evaluation. An HI titer of 6 log_2_ or above was applied as the criterion for the evaluation of H9N2 LPAIV vaccines [26]. The MDA 6 group satisfied this criterion and was completely protected against clinical disease; however, three of eight birds were positive in the CT, and all eight birds shed virus from the OP. The benchmark was established from studies of active vaccination, largely in SPF chickens, in which an HI titer serves as a correlate of a broader vaccine-induced response; passively acquired maternal IgY is accompanied by no such response, and the HI titer therefore represents only one measurable component of maternally acquired humoral immunity rather than a surrogate for the broader immune response induced by active vaccination. Consequently, it is advisable that the same numerical threshold should not be applied to actively and passively acquired immunity without qualification, and that the serological monitoring of broiler herds against this threshold should not be relied upon where the question of interest is transmission rather than disease. Under these circumstances, virological sampling of the oropharynx is the appropriate procedure.

This study had several limitations. No mock-challenged group was maintained under the same experimental conditions, so background effects unrelated to viral infection cannot be fully excluded; the challenge results should therefore be interpreted as comparisons among challenged birds with different pre-challenge MDA levels. A positive-control group with established active immunity was also not included, preventing direct comparison of MDA-mediated protection with active immunity under the same challenge conditions. With eight birds per group, the power to detect the observed difference in CT positivity between the extreme strata was approximately 40%; the trend across strata was significant, but no pairwise comparison was, and the CI of every PI value included zero; therefore, these point estimates should be regarded as indicating the direction and approximate magnitude of the effect rather than as precise estimates. The clinical and shedding results were more robust, the former because the trend was significant at *p* < 0.001, and the latter because all eight birds in all four groups were positive, which does not constitute a null result attributable to limited power. Birds were allocated to groups based on their measured titer rather than at random, with the consequence that any factor correlated with maternal antibody level is confounded with the exposure. Because group allocation was based on naturally occurring pre-challenge HI titers, potential confounding by maternal lineage or other egg-derived factors cannot be excluded. In addition, clinical assessment was not blinded, which may have introduced observer bias. These limitations should be considered when interpreting the observed association between MDA level and clinical outcome. All birds were challenged at a single age of 7 days by the intranasal route using a fixed high dose of 10^7.0^ EID_50_ of the Y280-lineage strain A21-MRA-003. Given the estimated MDA half-life of 3.2 days, MDA levels would be expected to decline further by 14 or 21 days of age, and the degree of protection observed at 7 days may therefore not be maintained at later ages. However, because challenge was performed only at 7 days of age, protection at later ages was not directly evaluated in the present study. In addition, the observed differences among clinical disease, CT virus detection, and OP shedding were determined using a single homologous Y280-lineage strain and may not necessarily apply to other H9N2 lineages, such as G1 or Korean/Y439. Challenge dose may also influence the apparent degree and anatomical distribution of protection; therefore, it remains to be determined whether the same pattern would be observed at lower challenge doses that may more closely approximate natural exposure conditions or following contact exposure. Transmission itself was not measured, and the inference that maternally protected birds are capable of sustaining transmission rests upon the prevalence and titer of shedding rather than upon an observed transmission event. Immunoglobulin isotypes were not measured in the serum or at the mucosal surfaces of our birds; therefore, the compositional explanation advanced above is derived from published isotype-transfer data [21] and from the established mucosal immunology of avian influenza [22,23,28]. This is consistent with our results but was not demonstrated here, and the measurement of lachrymal and tracheal IgA and IgY across MDA strata would permit it to be tested directly. The present challenge experiment was not designed to quantify economic losses or production performance. No mortality occurred in any challenge group, and body weight gain, feed conversion ratio, and flock-level performance were not measured. Therefore, the production and economic consequences of the observed clinical and virological outcomes cannot be determined from these data and should be evaluated in future field studies.

Notwithstanding these limitations, the results of the present study have several practical consequences for the control of H9N2 in commercial broilers. Raising breeder flock titers may have limited benefit for increasing progeny antibody levels, because the higher breeder titers observed in this study were not accompanied by proportional increases in progeny titers. Serological monitoring based solely on an HI threshold of 6 log_2_ may provide false reassurance because birds with detectable maternal antibody can still shed H9N2 virus. Accordingly, virological surveillance should complement clinical observation and serological monitoring, particularly when infection is suspected despite mild or absent clinical signs. In commercial settings, detection of H9N2 can inform veterinary investigation and prompt a review of farm biosecurity practices, including the movement of personnel and equipment, separation of flocks, and procedures intended to limit spread between poultry houses and farms. The appropriate response should be determined in consultation with the attending veterinarian and in accordance with applicable animal-health guidance. The present study did not test these interventions or directly measure bird-to-bird transmission; therefore, their effectiveness in reducing H9N2 spread and flock-level impact remains to be evaluated.

Any intervention intended to protect broilers against H9N2 influenza virus must be effective from the earliest stages of life, which restricts it to administration on the day of age or in ovo, and hence to platforms that may be applied in the hatchery during the same handling operation as is already employed for Marek’s disease vaccination. Vectored vaccines capable of single administration at the day of age have been described for H9N2 [31,32,33,34] including constructs antigenically matched to the Y280 lineage [25,34]. It would be advisable to evaluate whether any of them reduces oropharyngeal shedding, which is the endpoint at which the maternal antibody was shown to be ineffective.

Further studies incorporating the measurement of mucosal immunoglobulin isotypes, challenges at different ages and doses, heterologous challenges, and transmission experiments should be undertaken to fully characterize the protection available to commercial broilers and evaluate alternatives to reliance on maternal immunity.

## 5. Conclusions

The present study demonstrated that maternal antibodies to H9N2 in commercial broilers are transferred at a mean efficiency of 18.2%, that the amount transferred does not increase in proportion to the titer of the breeder flock, that it decays with a half-life of 3.2 days and falls below the 6 log_2_ benchmark within approximately three days of hatching, and that at the titers actually attained, it suppresses clinical disease and reduces enteric infection but does not reduce oropharyngeal shedding. Therefore, maternal antibodies alone are unlikely to provide sustained protection throughout the commercial production cycle. Under the challenge conditions tested, MDA did not prevent oropharyngeal virus shedding; however, the effect of MDA on bird-to-bird transmission remains to be determined. Consequently, control strategies for H9N2 in broilers should be evaluated against virological rather than clinical or serological endpoints and should be directed towards immunity generated in the chick itself from the earliest days of life.

## Figures and Tables

**Table 1:** Hemagglutination inhibition (HI) antibody titers to H9N2 in six commercial broiler breeder flocks and their day-old progeny, together with the estimated efficiency of maternal antibody transfer were determined.

Farm	Breeder Flock Age (Weeks)	Breeder HI Titer ^a^	Day-Old Progeny HI Titer ^a^	Transfer Efficiency (%) ^b^
Farm 1	33	8.7 ± 1.5	7.1 ± 1.7	33.0
Farm 2	34	9.3 ± 0.7	6.4 ± 0.9	13.4
Farm 3	43	10.3 ± 0.8	6.7 ± 1.1	8.2
Farm 4	30	10.1 ± 0.6	6.8 ± 0.8	10.2
Farm 5	32	9.5 ± 0.9	6.4 ± 0.8	11.7
Farm 6	31	7.4 ± 0.8	5.8 ± 0.7	33.0
Mean ± SD	34 ± 5	9.2 ± 1.1	6.7 ± 0.4	18.2 ± 11.5 ^c^

^a^ log_2_ HI titer, expressed as the mean ± standard deviation. The between-farm range was 2.9 log2 for the breeder herds and 1.3 log_2_ for the progeny. ^b^ Calculated as 100 × 2^(progeny log2 GMT − breeder log2 GMT)^. GMT: geometric mean titer. ^c^ Arithmetic mean of the six farm-level values; 95% confidence interval, 6.1–30.4%. The pooled value derived from the mean log_2_ decrement of 2.68 log_2_ was 15.6%. Pearson’s correlation between breeder titer and transfer efficiency, r = −0.894, *p* = 0.016; between breeder titer and progeny titer, r = 0.596, *p* = 0.212.

**Table 2:** Clinical signs, OP shedding, and CT infection at 5 days post-challenge in commercial broilers stratified by MDA titer and challenged at 7 days of age with 10^7.0^ EID50 of A21-MRA-003 by the intranasal route.

Group	MDA Titer at Challenge (log_2_)	No. of Birds	Clinical Signs ^a^ (% Positive)	OP Swab, log_10_ EID_50_/mL ^b^ (% Positive)	CT, log_10_ EID_50_/mL ^b^ (% Positive; 95% CI) ^c^	Absolute Risk Difference (Per-Centage Points)	PI (%) ^d^
MDA 0	0	8	8/8 (100)	6.1 ± 0.19 (100)	3.3 ± 1.19 (87.5; 47.3–99.7)	0(Reference)	0
MDA 2	2	8	8/8 (100)	5.9 ± 0.28 (100)	3.0 ± 0.63 (75.0; 34.9–96.8)	−12.5	14.3
MDA 4	4	8	7/8 (87.5)	6.0 ± 0.70 (100)	3.0 ± 1.10 (62.5; 24.5–91.5)	−25.0	28.6
MDA 6	6	8	0/8 (0)	6.4 ± 0.52 (100)	3.0 ± 1.15 (37.5; 8.5–75.5)	−50.0	57.1
MDA 0	0	8	8/8 (100)	6.1 ± 0.19 (100)	3.3 ± 1.19 (87.5; 47.3–99.7)	0(Reference)	0
MDA 2	2	8	8/8 (100)	5.9 ± 0.28 (100)	3.0 ± 0.63 (75.0; 34.9–96.8)	−12.5	14.3
MDA 4	4	8	7/8 (87.5)	6.0 ± 0.70 (100)	3.0 ± 1.10 (62.5; 24.5–91.5)	−25.0	28.6
MDA 6	6	8	0/8 (0)	6.4 ± 0.52 (100)	3.0 ± 1.15 (37.5; 8.5–75.5)	−50.0	57.1

^a^ Number of birds exhibiting clinical signs/total number of birds (%). b Cochran–Armitage test for trend across the ordered strata, Z = −4.40, *p* < 0.001; MDA 0 versus MDA 6, Fisher’s exact test, *p* = 0.0002. ^b^ Mean ± standard deviation of positive birds. OP titer, one-way ANOVA, F(3,28) = 1.71, *p* = 0.19. CT titer among positive birds: F(3,17) = 0.13, *p* = 0.94. ^c^ CT positivity, Cochran–Armitage test for trend Z = −2.16, *p* = 0.031; MDA 0 versus MDA 6, Fisher’s exact test *p* = 0.119; overall χ^2^ *p* = 0.183. CI = exact (Clopper − Pearson) confidence interval. ^d^ Protection index calculated against the MDA 0 group; confidence intervals were derived from the corresponding risk ratios.

**Table 3:** Published estimates of maternal antibody transfer efficiency for avian influenza virus in chickens, together with the methodological features associated with the differences between them.

Study	System	Assay	Source of Dam Titer	Age of Progeny at Sampling	Transfer Efficiency
Present study	Six commercial broiler-breeder farms, Korea (Y280 endemic)	HI (H9N2)	Measured, paired	Day of hatch	18.2 ± 11.5% (8.2–33.0%)
Gharaibeh et al. [15]	One commercial broiler-breeder flock, Jordan	ELISA (AIV)	Measured, paired	Day of hatch	19.5%
Hamal et al. [21]	Two meat lines, USA	ELISA (total IgY)	Measured, paired	3 days	≈30% for IgY; <1% for IgA and IgM
Mirzaie et al. [16]	Broiler farms, Qazvin Province, Iran	HI (H9N2)	Questionnaire and farm records	3 days	60.35%

**Table 4:** Half-life of maternal antibodies in commercial broiler chicks according to the pathogen. With the exception of the present study, all values were obtained within a single cohort under one protocol [17], which permitted ranking within the study.

Pathogen	Half-Life (Days)	Source
Chicken anaemia virus	7.0	[17]
Newcastle disease virus	6.3	
Avian encephalomyelitis virus	5.3	
Infectious bursal disease virus	5.1	
*Mycoplasma gallisepticum*	4.9	
Reovirus	4.7	
Avian influenza virus	4.2	
*Mycoplasma synoviae*	4.1	
Infectious bronchitis virus	3.9	
Infectious laryngotracheitis virus	3.8	
H9N2 avian influenza virus	3.2	Present study

## Data Availability

Data supporting the findings of this study are available from the corresponding author upon request. The data have not been deposited in a public repository owing to institutional policies and commercial confidentiality relating to the participating farms.

## Abbreviations

The following abbreviations are used in this manuscript:

AIV	Avian influenza virus
ANOVA	One-way analysis of variance
ARRIVE	Animal Research: Reporting of In Vivo Experiments
BSL-2	Biosafety level 2
CI	Confidence interval
CT	Cecal tonsil
DPC	Days post-challenge
EID50	50% egg infectious dose
GMT	Geometric mean titer
HA	Hemagglutinin
HI	Hemagglutination inhibition
IACUC	Institutional Animal Care and Use Committee
IBV	Infectious bronchitis virus
LPAIV	Low-pathogenic avian influenza virus
MDA	Maternally derived antibody
NA	Neuraminidase
OP	Oropharyngeal
PI	Protection index
SD	Standard deviation
SPF	Specific pathogen-free
